# Effect of carbon nanoparticle suspension injection versus indocyanine green tracer in guiding lymph node dissection during radical gastrectomy (FUTURE-01): a randomized clinical trial

**DOI:** 10.1097/JS9.0000000000003370

**Published:** 2025-09-04

**Authors:** Yusan Jin, Bingli Guo, Jing Li

**Affiliations:** a Department of Nuclear Medicine, The First Affiliated Hospital of Dalian Medical University, Dalian, China; b College of Laboratory Medicine, Dalian Medical University, Dalian, China; c Department of Cardiovascular Examination, The First Affiliated Hospital of Dalian Medical University, DaLian, China

*Dear Editor*,

We read with great interest the article titled “Effect of carbon nanoparticle suspension injection versus indocyanine green tracer in guiding lymph node dissection during radical gastrectomy (FUTURE-01): a randomized clinical trial” published in the *International Journal of Surgery*, authored by Tian *et al*[1]. This study is the first randomized controlled trial (RCT) to compare the efficacy of carbon nanoparticle suspension injection (CNSI) with indocyanine green (ICG) tracers in radical lymph node (LN) dissection during gastrectomy for gastric cancer. The rigorous design and significant findings of this study mark a milestone in the development of LN navigation technology for gastric cancer, and we sincerely commend this work. We hereby declare that this manuscript was translated and linguistic checked using artificial intelligence to ensure compliance with journal requirements. The TITAN Guideline checklist was submitted according to the requirements of the literature “Transparency In the reporting of Artificial Intelligence – The TITAN guideline”[2].

LN dissection is a critical component of gastrectomy for gastric cancer, and its quality directly affects patient prognosis and accuracy of pathological N staging^[3,4]^. Advances in intraoperative LN tracking technology are crucial for improving the quality of LN dissection and correcting postoperative N staging discrepancies[5]. CNSI and ICG have been widely used as novel LN tracers for intraoperative LN localization and postoperative pathological LN classification[6]. However, there is a lack of prospective randomized controlled clinical studies to thoroughly compare these two tracers^[7,8]^. Tian *et al*’s RCT fills this important gap, providing robust evidence-based support for selecting the optimal LN navigation technology in clinical practice.

The key strength of this study lies in its prospective, RCT design, which ensures the high reliability of the results. The research team enrolled 96 patients with potentially resectable gastric cancer and randomly assigned them to either the CNSI or ICG groups. This rigorous design minimized bias. The results clearly demonstrate that, compared with the ICG group, the CNSI group had a significantly increased total number of detected LNs, with an average of 69.8 and 53.6 detected LNs, respectively (*P* < 0.001). Notably, the CNSI group also detected a significantly higher number of micro-LNs than the ICG group (19.9 vs. 11.6, *P* = 0.001), which is crucial for improving the accuracy of pathological N staging, as the presence of micro-LNs helps to correct biases in N staging. Although there was no significant difference in the number of metastatic LNs between the two groups, CNSI-guided lymphangiography demonstrated superior sensitivity in detecting metastatic LNs compared with ICG (72.1% vs. 42.8%). Additionally, this article highlights that CNSI has lower clinical application costs and better clinical scalability, particularly in healthcare systems or hospitals with lower technological advancement levels.

The findings of this study have important clinical implications in gastric cancer surgery. A higher number of detected LNs, particularly micrometastatic LNs, can help more accurately assess tumor infiltration and LN metastasis status, thereby optimizing individualized treatment plans for patients. Previous studies have demonstrated that an increased number of detected LNs is associated with improved prognosis^[9–11]^. Therefore, CNSI is expected to enhance the accuracy of pathological staging in gastric cancer patients, avoid under-staging or excessive LN dissection, and provide patients with better long-term survival benefits. More importantly, studies have also confirmed that there are no significant differences between CNSI and ICG in terms of surgical complication rates, intraoperative blood loss, surgical duration, and postoperative recovery time, and no complications related to tracer injection were observed, fully demonstrating the safety of both LN navigation techniques in radical gastric cancer surgery. Given the cost-effectiveness and higher LN detection rate of CNSI, this tracer is likely to be the preferred option for LN dissection.

In summary, Tian *et al* provided the first direct comparative evidence for the application of CNSI and ICG in gastric cancer LN dissection in a high-quality RCT. The findings of this study support CNSI as a superior and more cost-effective LN navigation tool, which is expected to significantly improve the accuracy of gastric cancer LN dissection and refinement of pathological N staging, thereby improving patient management and prognosis. We believe that this study will have a profound impact in the field of gastric cancer surgery.

## Data Availability

No datasets were generated or analysed during the current study.

## Research registration unique identifying number (UIN)

Not applicable. This study does not involve human participants and therefore does not require registration.
